# Use of magnetic resonance targeting to steer ov-loaded cell-based therapies to tumor sites *in vivo*

**DOI:** 10.1186/2051-1426-3-S2-P339

**Published:** 2015-11-04

**Authors:** Munitta Muthana, Aneurin Kennerley, Emer Murphy, Russell Hughes, Joe Conner, Fiona Wright, Mark Lythgoe, Jon Dobson, Jim Wild, Claire Lewis

**Affiliations:** 1 University of Sheffield, Sheffield, UK; 2 Virttu Biologics, Glasgow, UK; 3 University College London, London, UK; 4 University of Florida, Florida, FL, USA

## Background

Despite considerable progress in the development of cell-based therapies, targeted delivery to specific tissues - particularly those deep in the body where direct injection is not possible - has been problematic. Here we show that tumour-conditioned macrophages infected with oncolytic HSV Seprehvir display a classic activated (M1) profile characterized by the expression of pro-inflammatory factors such as iNOS, IL-6, IL-8 and tumor necrosis factor-α (TNF-α). Furthermore, the M1 macrophages can be magnetically labeled using super-paramagnetic iron oxide nanoparticles (SPIOs) and then steered from the bloodstream into deep target tissues using pulsed magnetic-field gradients inherent to all magnetic resonance imaging systems (MRI). We have called this approach magnetic resonance targeting (MRT) and have used it to deliver a cell-based oncolytic virotherapy.

## Methods

SPIO-loaded macrophages, armed with Seprehvir, were administered intravenously to mice bearing orthotopic primary and metastatic (lung) prostate tumors. Mice were positioned in the MRI scanner and pulsed magnetic field gradients were applied for 1 hour, to steer the magnetic cells towards the target site by MRT (MRT). In control conditions mice were exposed to the static magnetic field of the scanner but gradients were not pulsed (No MRT).

## Results

MRI steering significantly increased uptake of SPIO-loaded macrophages in primary prostate tumours (MRT: 42.2%±2.5 vs. No MRT: 7.17%±0.8, *p*=0.0001) and pulmonary metastasis (MRT: 17.7%±4 vs. No MRT:4.4%±2.6, *p*=0.01) as assessed by magnetic *relaxometry* and MRI and post-mortem by flow cytometry and histology. Crucially, this increased uptake of magnetic, Seprehvir-armed macrophages led to marked tumour shrinkage and reduced metastatic burden.

## Conclusions

Our study demonstrates the potential for clinical MRI scanners not only to image such magnetically labeled cells after their injection into the body, but also to steer non-invasively, therapeutically-loaded cells specifically to one or more tumors within the body.

